# Associations of inflammation-related nutritional and metabolic status indices CAR and CTI with 90-day unfavorable functional outcomes in patients with acute ischemic stroke

**DOI:** 10.3389/fnut.2026.1790922

**Published:** 2026-03-23

**Authors:** Xijun Liu, Xiaojuan Zheng, Zeru Chen, Haiwei Chen, Mingxi Xie, Jing Zhang

**Affiliations:** 1 Department of Laboratory Medicine, Yuebei People's Hospital, Shantou University Medical College, Shaoguan, China; 2 The Second Department of Infectious Disease, Shanghai Fifth People's Hospital, Fudan University, Shanghai, China; 3 Center of Community-Based Health Research, Fudan University, Shanghai, China; 4 Guangzhou Medical University, Guangzhou, Guangdong, China; 5 Southern Medical University, Guangzhou, Guangdong, China

**Keywords:** acute ischemic stroke, CAR, cardiometabolic risk, CTI, prognosis

## Abstract

**Background:**

Composite indices integrating inflammation, nutritional reserve, and metabolic status may improve early prognostic assessment after acute ischemic stroke (AIS). This study examined the associations of the C-reactive protein-to-albumin ratio (CAR) and the CRP–triglyceride–glucose index (CTI) with 90-day functional outcomes.

**Methods:**

A Korean single-center stroke registry (2010–2016) was analyzed (*n* = 1,484). CAR and CTI were calculated from admission laboratory tests. The primary endpoint was 90-day unfavorable functional outcome defined as modified Rankin Scale 3–6. Associations were examined using multivariable logistic regression with standardized exposures; restricted cubic splines assessed non-linearity. Prognostic performance was evaluated using receiver operating characteristic analysis and a parsimonious model derived with penalized regression. Internal validation was performed using repeated 10-fold cross-validation, and model calibration was assessed using a calibration plot, calibration intercept, calibration slope, and Brier score.

**Results:**

Unfavorable outcomes occurred in 414 patients (27.9%). After full adjustment, higher CAR (OR 1.25, 95% CI 1.09–1.43) and higher CTI (OR 1.38, 95% CI 1.19–1.60) were independently associated with unfavorable outcome. CTI showed an approximately linear risk gradient, whereas CAR showed evidence of a non-linear association. As single predictors, CAR and CTI yielded AUCs of 0.656 and 0.651 and outperformed albumin, triglycerides, and fasting plasma glucose. A six-variable model including sex, age, body mass index, admission NIHSS, CAR, and CTI achieved an apparent AUC of 0.837. Internal validation showed stable performance, with a mean cross-validated AUC of 0.831, mean Brier score of 0.142, calibration intercept of 0.023, and calibration slope of 1.018.

**Conclusion:**

CAR and CTI, derived from routine admission laboratory tests, were associated with 90-day functional outcomes after acute ischemic stroke and may aid early prognostic assessment. The derived six-variable model showed acceptable internal performance, but should be considered exploratory pending external validation.

## 1 Introduction

Acute ischemic stroke (AIS) continues to generate profound mortality and long-lasting disability worldwide, translating into substantial societal and healthcare costs (1). Notably, even with the rapid evolution of endovascular interventions and standardized acute care pathways, recovery trajectories after AIS remain strikingly diverse (2). This variability underscores the clinical need for early risk profiling so that treatment planning can be better informed, rehabilitation intensity can be prioritized appropriately, and secondary prevention can be tailored to the individual.

Growing evidence suggests that poor outcomes after AIS arise from a network of processes that extend beyond the initial ischemic insult. A systemic inflammatory surge and disturbed metabolic regulation are increasingly recognized as key drivers of secondary brain injury, determinants of neurorestorative capacity, and promoters of post-stroke medical complications (3–5). Elevated C-reactive protein (CRP), for instance, is frequently observed in the acute phase and has been linked to blood–brain barrier compromise, lesion progression, and limited functional recovery (6–8). In addition, reduced physiological reserve—often reflected by low serum albumin—together with metabolic abnormalities such as dysglycemia and lipid disturbances, can heighten vulnerability to infections and venous thromboembolism and may also impair neuronal survival and plasticity (9–11). Despite these biological insights, prognostic evaluation in AIS is still dominated by conventional clinical and imaging predictors, including age, the National Institutes of Health Stroke Scale (NIHSS), and neuroimaging features (12–14). In contrast, readily obtainable serum markers that summarize multiple pathophysiological domains are not yet routinely incorporated into early risk stratification.

Composite biomarker indices may offer an efficient solution because they integrate signals from interconnected pathways within a single metric. CAR jointly captures acute-phase inflammation and nutritional/anti-inflammatory reserve, thereby providing a pragmatic readout of the imbalance between inflammatory activity and host resilience (15). CAR has been reported to carry prognostic information in several clinical contexts, such as biliary inflammatory conditions, community-acquired pneumonia, heart failure, and certain malignancies (16–18). Extending this concept, CTI combines CRP with triglycerides and fasting glucose to reflect coupled inflammation–metabolic stress and has shown promise in cardiovascular risk prediction (19). However, evidence in AIS remains insufficient, particularly from prospective cohorts, regarding whether CAR and CTI independently relate to short-term functional outcomes, whether their associations follow a graded pattern, and whether they improve prediction beyond established clinical models.

To address these gaps, a secondary analysis of a single-center prospective stroke registry was conducted to examine the associations of baseline CAR and CTI with 90-day functional outcomes after AIS. The primary objective was to determine whether CAR and CTI were independently associated with unfavorable outcome and whether they provided incremental prognostic value beyond established clinical predictors. Multivariable logistic regression with hierarchical adjustment, restricted cubic spline modeling, prespecified interaction analyses, and ROC/AUC assessment were applied. In addition, machine-learning–assisted feature selection using penalized regression was used to derive a compact exploratory prognostic model, and its discrimination, calibration, and potential clinical utility were evaluated.

## 2 Materials and methods

### 2.1 Data source and ethical approval

Data were obtained from a prospectively maintained, single-center stroke registry in Korea. The registry and its data collection procedures have been described previously by Kang et al. (20), and the de-identified dataset used in the present analysis was accessed under the data-use terms of that report. The original registry study complied with the Declaration of Helsinki and was approved by the Institutional Review Board of Seoul National University Hospital, with a waiver of informed consent (IRB No. 1009-062-332). All records analyzed were anonymized and contained no personally identifiable information.

Kang et al. (20) publication is distributed under the Creative Commons Attribution (CC BY) license, under which the material may be reused in any format as long as proper attribution to the authors and source is provided. To ensure ethical and regulatory compliance, all records used in the present study were fully anonymized and contained no personally identifiable information.

### 2.2 Study population and exclusion criteria

**Figure 1:**
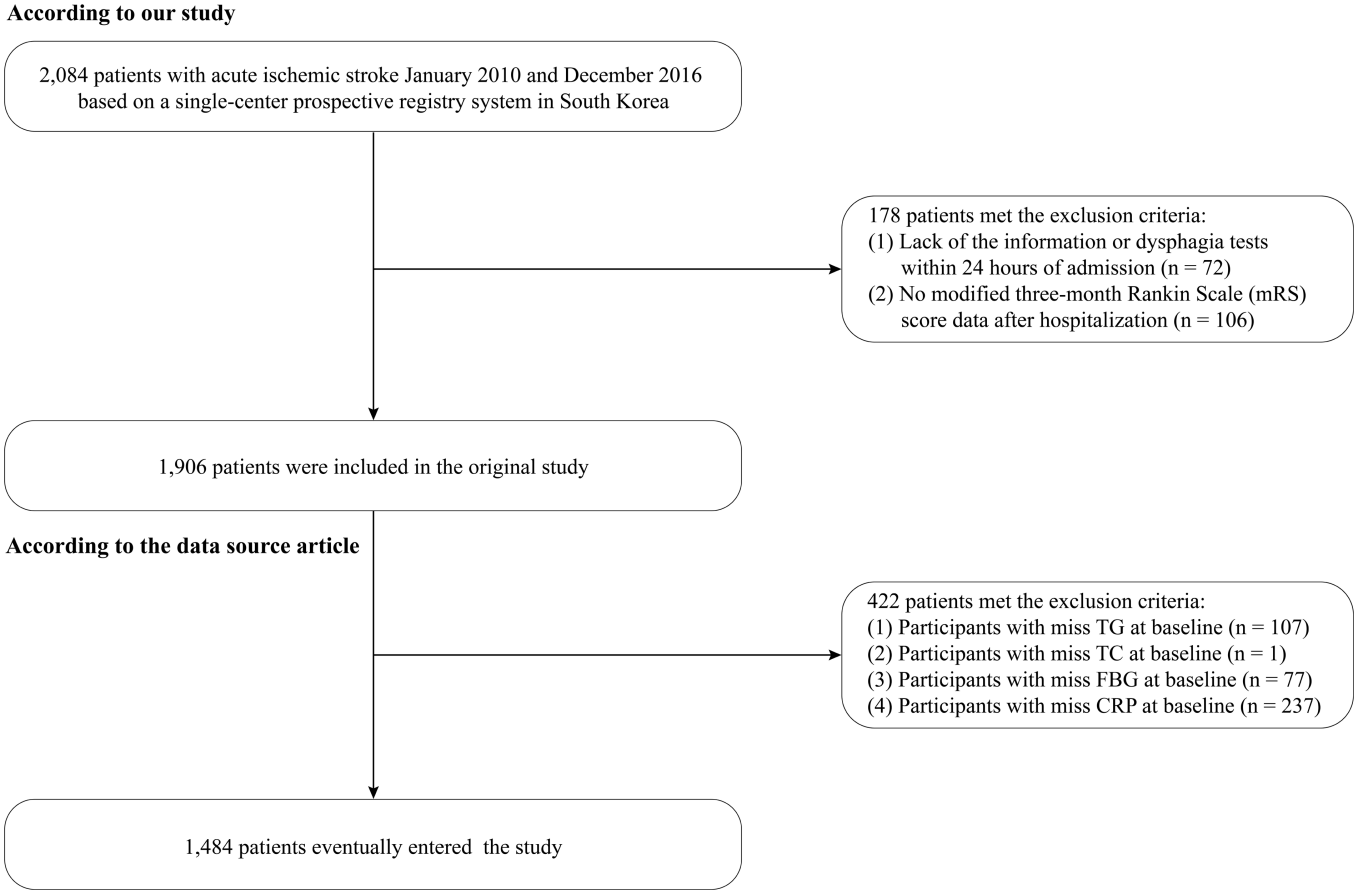
Flow diagram of participant selection for the present study.

Between January 2010 and December 2016, a total of 2,084 patients with acute ischemic stroke were initially screened from the source registry. Of these, 72 patients were excluded because dysphagia screening within 24 h of admission was unavailable, and 106 patients were excluded because 3-month modified Rankin Scale (mRS) data were missing, leaving 1,906 participants in the original registry cohort (21). For the present secondary analysis, an additional 422 patients were excluded because baseline laboratory variables required for the calculation of CAR, CTI, or key covariates were missing. The final analytic cohort therefore comprised 1,484 participants (Figure 1).

### 2.3 Exposures and outcomes

CAR and the CTI index were prespecified as the main exposure measures. CAR was computed as CRP divided by ALB (22). CTI was calculated following a published approach (23) as: CTI = 0.412 × ln[CRP (mg/L)] + (ln[TG (mg/dL)] + ln[FPG (mg/dL)])/2.

The primary outcome was 90-day post-stroke functional status. Modified Rankin Scale (mRS) scores were collected at follow-up through clinic visits or standardized telephone interviews. For analysis, patients were classified as having a favorable outcome (mRS 0–2) or an unfavorable outcome (mRS 3–6) (24, 25).

### 2.4 Clinical and laboratory variables

Baseline covariates were extracted from the registry and medical records (26) and included demographics (age, sex, body mass index, and smoking status), relevant medical history (hyperlipidemia, diabetes mellitus, atrial fibrillation, coronary heart disease, and prior transient ischemic attack), and stroke severity measured by the NIHSS at both admission and discharge. Stroke etiology was coded using predefined categories (large-artery atherosclerosis, small-vessel occlusion, cardioembolism, other determined causes, and undetermined causes). Nutritional status was assessed with the Nutritional Risk Index (NRI) and analyzed by risk strata.

Routine laboratory variables were extracted from the source registry and electronic medical records and reflected tests obtained during the initial hospitalization in patients admitted within 7 days of stroke onset. Because this was a secondary analysis of a de-identified dataset, the exact interval between stroke onset and blood sampling for each analyte was not available. Fasting status was also not uniformly documented for the laboratory measurements. The laboratory panel comprised hematologic indices (white/red blood cell counts, hemoglobin, red cell distribution width, and platelet count), lipid parameters (TC, TG, HDL-C, and LDL-C), renal function markers (blood urea nitrogen and creatinine), glycemic markers (FPG and HbA1c), and inflammation/protein-related measures (CRP, ALB, and total protein).

Covariate adjustment was restricted to variables available in the shared de-identified dataset. Information on pre-stroke functional status, acute infection status, reperfusion therapies, and exact analyte-specific laboratory sampling time was not consistently available for inclusion in the multivariable models.

### 2.5 Statistical analysis

Statistical procedures were carried out in R (v3.4.3) and FreeStatistics (v2.4.0). All tests were two-tailed, with *p* < 0.05 indicating statistical significance. Participant characteristics are summarized using mean ± SD for approximately normally distributed continuous variables and median (IQR) for skewed variables; categorical data are presented as *n* (%). For comparability, CAR and CTI were standardized to z-scores prior to modeling.

Patients with missing values in variables required for the calculation of CAR, CTI, or other essential model inputs were excluded before analysis. Among the remaining analytic cohort, missing values in other covariates were handled using multiple imputation rather than simple complete-case analysis. This approach was used to reduce bias and loss of information associated with excluding all patients with partial missingness.

The primary endpoint was an unfavorable functional outcome at 90 days (mRS ≥ 3). Associations of CAR and CTI with the endpoint were evaluated in separate multivariable logistic regression models, reporting odds ratios (ORs) and 95% confidence intervals (CIs). Multicollinearity among covariates included in the fully adjusted Model 3 was assessed using variance inflation factors. For categorical variables with more than one degree of freedom, generalized variance inflation factors (GVIFs) and adjusted GVIF^(1/(2 × Df)) values were reported. Confounding control was implemented through a hierarchical adjustment approach using three nested models in which covariates were added sequentially to examine the stability of estimates. To explore potential departures from linearity, restricted cubic spline terms were fitted within the logistic models. When evidence of non-linearity was observed for CAR, a two-piecewise logistic regression model with the breakpoint fixed at 1.32 was fitted as a secondary exploratory analysis.

Prespecified subgroup analyses were conducted across strata defined by age, sex, and major comorbid conditions, and interaction terms were introduced in the fully adjusted model to formally assess effect modification. Discrimination was evaluated with receiver operating characteristic (ROC) curves and the area under the curve (AUC). The added predictive value of CAR and CTI beyond a conventional clinical model was further assessed using net reclassification improvement (NRI) and integrated discrimination improvement (IDI) (27).

To identify the most informative clinical predictors, Boruta was first used as a robustness-oriented feature-screening method to assess the relevance of candidate variables to 90-day unfavorable outcome. Least absolute shrinkage and selection operator (LASSO) regression was then applied to derive a parsimonious prognostic model by shrinking less informative coefficients toward zero (27). A compact prognostic model comprising sex, age, BMI, NIHSS, CAR, and CTI was then constructed. Model discrimination was summarized by the area under the ROC curve (AUC), and potential clinical benefit was examined using decision-curve analysis (DCA). Internal validation was performed using repeated 10-fold cross-validation. Model calibration was assessed using a calibration plot, calibration intercept, calibration slope, Brier score, and calibration error measures. Because no independent external dataset was available for the present secondary analysis, validation was limited to internal cross-validation.

## 3 Results

### 3.1 Baseline profile stratified by 3-month mRS

A total of 1,484 patients were included; 1,070 (72.1%) achieved a favorable 3-month outcome (mRS 0–2) and 414 (27.9%) had an unfavorable outcome (mRS 3–6) (Table 1). CTI differed markedly between groups, with greater values observed in the unfavorable group (*p* < 0.001).

**Table 1:** Baseline characteristics according to 3-month outcomes.

Variables	Total (*n* = 1,484)	Favorable outcome (*n* = 1,070)	Unfavorable outcome (*n* = 414)	*P*-value
CTI, Median (IQR)	1.6 (−1.7, 5.9)	0.9 (−2.0, 4.6)	4.0 (0.3, 9.8)	**<0.001**
Demographics				
Sex [Male, *n* (%)]	912 (61.5)	703 (65.7)	209 (50.5)	**<0.001**
Age, *n* (%)				**<0.001**
<60	324 (21.8)	266 (24.9)	58 (14)	
60–70	393 (26.5)	307 (28.7)	86 (20.8)	
70–80	528 (35.6)	369 (34.5)	159 (38.4)	
>=80	239 (16.1)	128 (12)	111 (26.8)	
BMI, Mean ± SD	23.48 ± 3.28	23.74 ± 3.13	22.81 ± 3.55	**<0.001**
Smoking, *n* (%)	599 (40.4)	468 (43.7)	131 (31.6)	**<0.001**
Medical history, *n* (%)				
Hyperlipidemia	551 (37.1)	413 (38.6)	138 (33.3)	0.060
DM	456 (30.7)	309 (28.9)	147 (35.5)	**0.013**
AF	312 (21.0)	186 (17.4)	126 (30.4)	**<0.001**
CHD	175 (11.8)	129 (12.1)	46 (11.1)	0.613
TIA	308 (20.8)	188 (17.6)	120 (29)	**<0.001**
Clinical status on admission				
Initial NIHSS score Median (IQR)	3.0 (1.0, 7.0)	2.0 (1.0, 4.0)	9.0 (4.0, 16.0)	**<0.001**
Discharge NIHSS, Median (IQR)	2.0 (0.0, 4.0)	1.0 (0.0, 3.0)	6.0 (2.0, 13.0)	**<0.001**
Stroke etiology, *n* (%)				**<0.001**
LAA	490 (33.0)	362 (33.8)	128 (30.9)	
SVO	283 (19.1)	236 (22.1)	47 (11.4)	
CE	373 (25.1)	241 (22.5)	132 (31.9)	
Other determined	124 (8.4)	70 (6.5)	54 (13)	
Undetermined	213 (14.4)	161 (15)	52 (12.6)	
NRI, Mean ± SD	105.21 ± 9.66	106.57 ± 8.79	101.67 ± 10.85	**<0.001**
Nutritional risk index, *n* (%)				**<0.001**
No risk	1,083 (73.0)	835 (78)	248 (59.9)	
Mild risk	92 (6.2)	60 (5.6)	32 (7.7)	
Moderate risk	278 (18.7)	168 (15.7)	110 (26.6)	
Severe risk	31 (2.1)	7 (0.7)	24 (5.8)	
Laboratory parameters				
WBC (10^9/L, Mean ± SD)	8.20 ± 2.96	8.02 ± 2.71	8.69 ± 3.49	**<0.001**
RBC (10^9/L, Mean ± SD)	4.33 ± 0.63	4.39 ± 0.61	4.19 ± 0.66	**<0.001**
HGB (g/L, Mean ± SD)	13.50 ± 1.98	13.71 ± 1.87	12.95 ± 2.14	**<0.001**
RDW (%, Mean ± SD)	13.38 ± 1.53	13.24 ± 1.31	13.75 ± 1.93	**<0.001**
PLT (10^9/L, Mean ± SD)	225.24 ± 69.79	225.88 ± 66.50	223.57 ± 77.70	0.568
TC (mg/dL, Mean ± SD)	180.66 ± 43.95	182.67 ± 43.34	175.47 ± 45.15	**0.005**
TG (mg/dL, Mean ± SD)	109.71 ± 54.58	112.62 ± 55.99	102.18 ± 50.03	**<0.001**
HDL-C (mg/dL, Mean ± SD)	46.63 ± 13.59	46.76 ± 13.37	46.31 ± 14.15	0.564
LDL-C (mg/dL, Mean ± SD)	108.83 ± 38.39	109.83 ± 37.63	106.24 ± 40.21	0.106
BUN (ummol/L, Mean ± SD)	17.46 ± 8.89	17.25 ± 8.52	17.99 ± 9.76	0.150
Creatinine [mg/dL, Median (IQR)]	0.9 (0.7, 1.1)	0.9 (0.8, 1.1)	0.8 (0.7, 1.0)	**0.012**
ALB (g/dL, Mean ± SD)	4.03 ± 0.42	4.09 ± 0.38	3.87 ± 0.47	**<0.001**
Total protein (g/dL, Mean ± SD)	7.02 ± 0.59	7.05 ± 0.57	6.95 ± 0.63	**0.002**
FPG (mg/dL, Mean ± SD)	106.58 ± 38.57	102.78 ± 32.60	116.40 ± 49.57	**<0.001**
HbA1c (%, Mean ± SD)	5.24 ± 2.64	5.19 ± 2.60	5.36 ± 2.73	0.266
CRP [mg/L, Median (IQR)]	1.6 (0.6, 5.3)	1.3 (0.6, 3.7)	3.2 (1.1, 15.8)	**<0.001**
CTI, *n* (%)				**<0.001**
Q1	371 (25.0)	309 (28.9)	62 (15)	
Q2	371 (25.0)	290 (27.1)	81 (19.6)	
Q3	371 (25.0)	267 (25)	104 (25.1)	
Q4	371 (25.0)	204 (19.1)	167 (40.3)	
CAR, *n* (%)				**<0.001**
Q1	371 (25.0)	310 (29)	61 (14.7)	
Q2	370 (24.9)	288 (26.9)	82 (19.8)	
Q3	371 (25.0)	269 (25.1)	102 (24.6)	
Q4	372 (25.1)	203 (19)	169 (40.8)	

CTI, C-reactive protein-triglyceride-glucose index; BMI, body mass index; DM, diabetes mellitus; AF, atrial fibrillation; CHD, coronary heart disease; NIHSS, National Institutes of Health Stroke Scale score; TIA, transient ischemic attack; LAA, large artery atherosclerosis; SVO, small vessel occlusion; CE, cardiac embolism; NRI, nutritional risk index; WBC, white blood cell; RBC, red blood cell; HGB, hemoglobin; RDW, red cell distribution width; PLT, platelets; ALB, albumin; FPG, fasting plasma glucose; HBA1c, hemoglobin Alc; HDL-C, high-density lipoprotein cholesterol; LDL-C, low-density lipoprotein cholesterol; CRP, C-reactive protein; TC, total cholesterol; BUN, blood urea nitrogen; TG, triglycerides. *P*-value less than 0.05 is expressed in bold.

Between-group imbalances were noted in sex and age composition, BMI, and smoking status. The unfavorable group showed a higher prevalence of diabetes, atrial fibrillation, and prior TIA, whereas hyperlipidemia and coronary heart disease were similar across groups. NIHSS scores at both admission and discharge were higher among patients with unfavorable outcomes. Etiologic subtype distributions also diverged (*p* < 0.001), characterized by a relative enrichment of cardioembolism and “other determined etiology” and a relative depletion of small-vessel occlusion.

Nutritional assessment indicated a lower NRI and a shifted nutritional risk stratification in the unfavorable group (*p* < 0.001). In laboratory profiles, WBC, RDW, fasting plasma glucose, and CRP were increased, while RBC, hemoglobin, albumin, and total protein were decreased (all *p* ≤ 0.002). Total cholesterol and triglycerides were lower (*p* = 0.005; *p* < 0.001), and creatinine was slightly reduced; platelet count, HDL-C, LDL-C, BUN, and HbA1c were comparable (all *p* > 0.05).

In quartile analyses, CTI and CAR strata were associated with 3-month outcomes (both *p* < 0.001). Patients with unfavorable outcomes were more frequently classified into Q4 (CTI: 40.3% vs. 19.1%; CAR: 40.8% vs. 19.0%). A descriptive comparison between the final analytic cohort and the 422 patients additionally excluded because of missing key laboratory variables is presented in Supplementary Table S3.

### 3.2 Boruta-based feature screening

**Figure 2:**
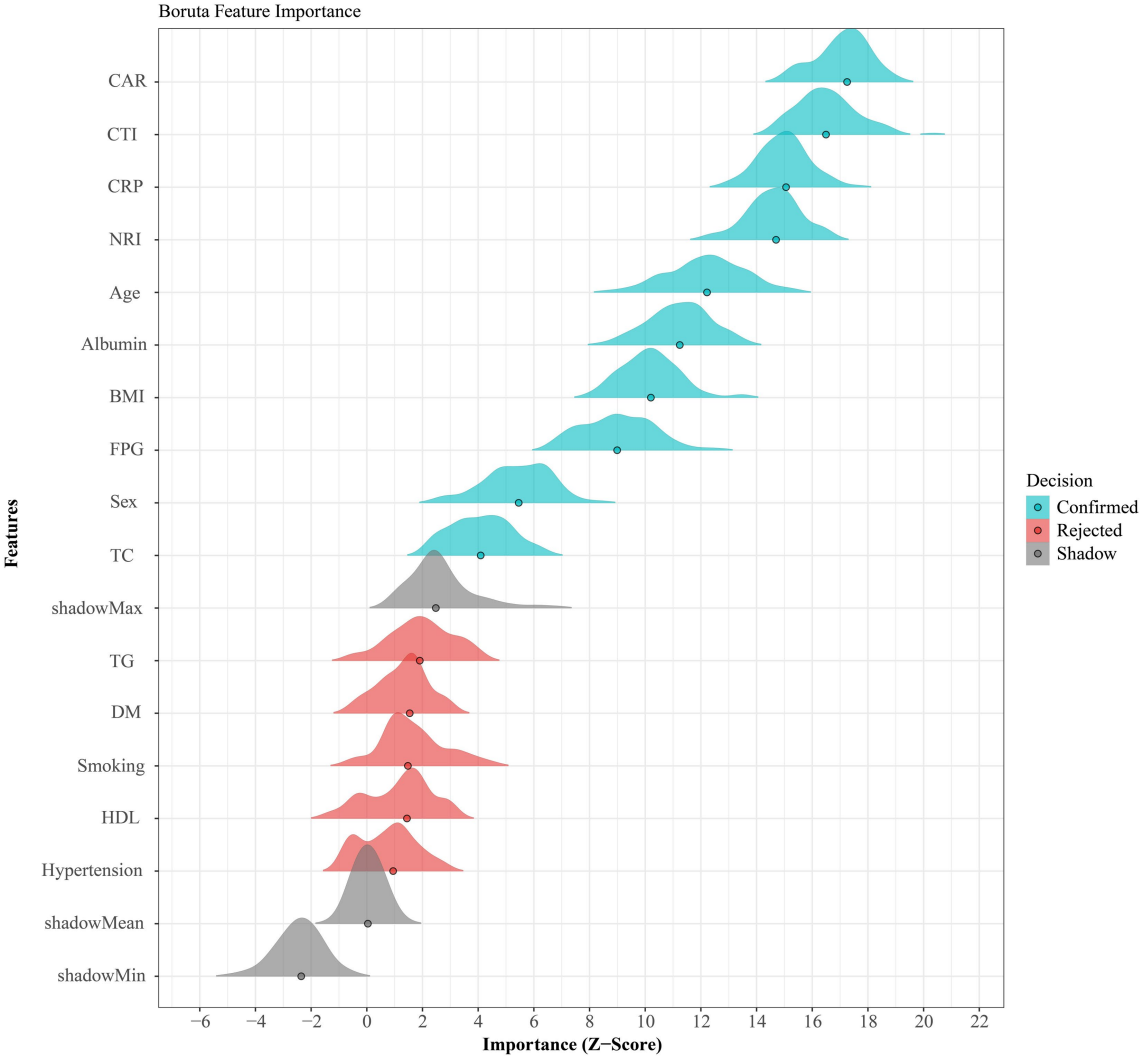
Boruta-based feature importance for predicting 90-day unfavorable functional outcome after AIS.

Boruta screening (Figure 2) prioritized CAR and CTI as the most stable predictors across 500 iterations. Age, sex, BMI, and smoking status were retained *a priori* for subsequent modeling irrespective of their Boruta ranking.

### 3.3 Associations of CAR and CTI with 90-day unfavorable outcomes in AIS

No evidence of problematic multicollinearity was observed in the fully adjusted models. In the CAR model, adjusted GVIF^(1/(2 × Df)) values ranged from 1.033 to 1.497, whereas in the CTI model they ranged from 1.033 to 1.496 (Supplementary Tables S1, S2). Because adjusted GVIF^(1/(2 × Df)) is intended to facilitate comparison across terms of different dimensionality, and all observed values were close to 1, substantial multicollinearity was considered unlikely.

Multivariable logistic models showed that CAR and CTI were each independently related to an unfavorable 90-day functional outcome in AIS (Table 2), and the direction of effect was maintained after stepwise adjustment. In the fully adjusted specification (Model 3), controlling for demographic factors, vascular comorbidities, stroke severity, nutritional status, and laboratory covariates, CAR (OR 1.25, 95% CI 1.09–1.43; *p* = 0.002) and CTI (OR 1.38, 95% CI 1.19–1.60; *p* < 0.001) remained associated with increased odds of poor outcome.

**Table 2:** Association of CAR and CTI with 90-day unfavorable outcomes follow AIS in different models.

Characteristic	Model 1			Model 2			Model 3		
	OR (95%CI)	*p*-value	*E*-value	OR (95%CI)	*P*-value	*E*-value	OR (95%CI)	*P*-value	*E*-value
Continuous CAR	1.51 (1.33 ~ 1.71)	<0.001	2.388	1.45 (1.28 ~ 1.64)	<0.001	2.258	1.25 (1.09 ~ 1.43)	0.002	1.809
Quartiles CAR									
Q1	Ref.			Ref.			Ref.		
Q2	1.45 (1.00 ~ 2.09)	0.049	2.258	1.49 (1.02 ~ 2.17)	0.038	2.344	1.41 (0.92 ~ 2.17)	0.113	2.170
Q3	1.93 (1.35 ~ 2.75)	<0.001	3.270	1.88 (1.30 ~ 2.71)	0.001	3.166	1.62 (1.06 ~ 2.48)	0.026	2.622
Q4	4.23 (3.01 ~ 5.96)	<0.001	7.926	4.11 (2.89 ~ 5.84)	<0.001	7.685	2.37 (1.54 ~ 3.63)	<0.001	4.171
*P* for trend	1.61 (1.45 ~ 1.80)	<0.001	2.601	1.59 (1.42 ~ 1.77)	<0.001	2.559	1.31 (1.15 ~ 1.50)	<0.001	1.947
Continuous CTI	1.77 (1.57 ~ 1.99)	<0.001	2.937	1.73 (1.53 ~ 1.95)	<0.001	2.853	1.38 (1.19 ~ 1.60)	<0.001	2.104
Quartiles CTI									
Q1	Ref.			Ref.			Ref.		
Q2	1.39 (0.96 ~ 2.01)	0.078	2.126	1.48 (1.02 ~ 2.16)	0.040	2.322	1.40 (0.91 ~ 2.15)	0.125	2.148
Q3	1.94 (1.36 ~ 2.77)	<0.001	3.29	1.93 (1.34 ~ 2.77)	<0.001	3.269	1.67 (1.10 ~ 2.54)	0.017	2.727
Q4	4.08 (2.90 ~ 5.74)	<0.001	7.624	4.06 (2.86 ~ 5.78)	<0.001	7.584	2.32 (1.51 ~ 3.54)	<0.001	4.069
*P* for trend	1.60 (1.44 ~ 1.79)	<0.001	2.579	1.58 (1.42 ~ 1.77)	<0.001	2.537	1.31 (1.15 ~ 1.50)	<0.001	1.947

Model 1: crude model.

Model 2 adjusted for sex, age.

Model 3 adjusted for Model 2 plus PLT, BMI, DM, Hypertension, Smoking, AF, Creatinine, TC, TG, HDL-C. Initial NIHSS, CHD, Hb1Ac, Stroke etiology.

CTI, C-reactive protein-triglyceride-glucose index; CAR, C-reactive protein/albumin ratio; BMI, body mass index; DM, diabetes mellitus; AF, atrial fibrillation; CHD, coronary heart disease; NIHSS, National Institutes of Health Stroke Scale score; PLT, platelets; HBA1c, hemoglobin Alc; HDL-C, high-density lipoprotein cholesterol; TC, total cholesterol; TG, triglycerides. OR: odd ratio. CI: confidence interval.

Across quartiles, a graded pattern was observed: relative to the lowest quartile, the highest quartile was linked to higher odds for both CAR (OR 2.37, 95% CI 1.54–3.63; *p* < 0.001) and CTI (OR 2.32, 95% CI 1.51–3.54; *p* < 0.001), with significant trend tests (both *P* for trend <0.001).

In the fully adjusted models, the *E*-values were 1.81 for continuous CAR and 2.10 for continuous CTI; for the highest-versus-lowest quartile comparisons, the corresponding *E*-values were 4.17 and 4.07. These estimates indicate that fully explaining the observed associations would require an unmeasured confounder with strong links to both the exposure and the outcome.

### 3.4 Non-linear associations of CAR and CTI with 90-day unfavorable outcomes

**Figure 3:**
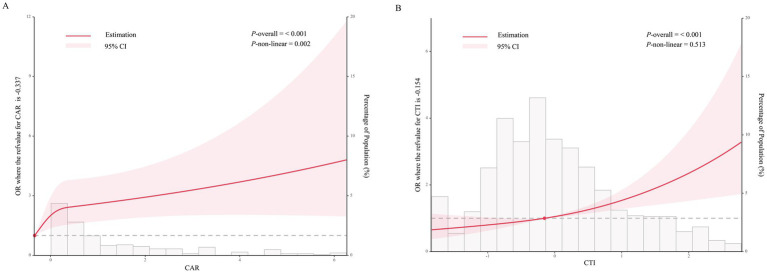
Restricted cubic spline analyses of the dose–response associations of CAR and CTI with 90-day unfavorable outcome. **(A)** Restricted cubic spline curve for the association between CAR and the study outcome. **(B)** Restricted cubic spline curve for the association between CTI and the study outcome. The solid red lines indicate the estimated odds ratios, and the shaded areas represent 95% confidence intervals. Histograms show the distribution of the study population. The reference values were −0.337 for CAR and −0.154 for CTI.

As shown in Figure 3, restricted cubic splines were used to examine departures from linearity. CTI showed an approximately linear relationship with the risk of 90-day unfavorable outcome, whereas CAR showed evidence of non-linearity (*P* for non-linearity = 0.002). In exploratory analysis, a two-piecewise logistic regression model with the breakpoint fixed at 1.32 was fitted. A total of 1,400 patients had CAR < 1.32, among whom 370 experienced an unfavorable outcome, whereas 84 had CAR ≥ 1.32, among whom 44 experienced an unfavorable outcome. Below the breakpoint, CAR was associated with higher odds of unfavorable outcome (OR 3.07, 95% CI 1.74–5.39; *p* = 0.0001), whereas above the breakpoint the estimate was less precise and did not reach statistical significance (OR 1.595, 95% CI 0.98–2.60; *p* = 0.0601) (Table 3). The log-likelihood ratio test supported non-linearity (*p* = 0.03). Because relatively few patients were in the higher CAR range, the piecewise estimate in this segment should be interpreted cautiously. The distribution of CAR values is shown in Supplementary Figure S3.

**Table 3:** Threshold effect analysis of CAR on 90-day unfavorable outcomes using two-piecewise linear regression.

Variables	Total population	Unfavorable outcome	mRS	
			Adjusted OR (95% CI)	*P-*value
Inflection point of CAR	1,484	414	1.32	
CAR < 1.32	1,400	370	3.07 (1.74, 5.39)	0.0001
CAR ≥ 1.32	84	44	1.595 (0.98, 2.60)	0.0601
*P* for log-likelihood ratio test	–	–	0.030	

Adjusted for Sex, Age, PLT, BMI, DM, Hypertension, Smoking, AF, Creatinine, TC, TG, HDL-C. Initial NIHSS, CHD, Hb1Ac, Stroke etiology.

### 3.5 Subgroup analyses of the CAR– and CTI–outcome associations

**Figure 4:**
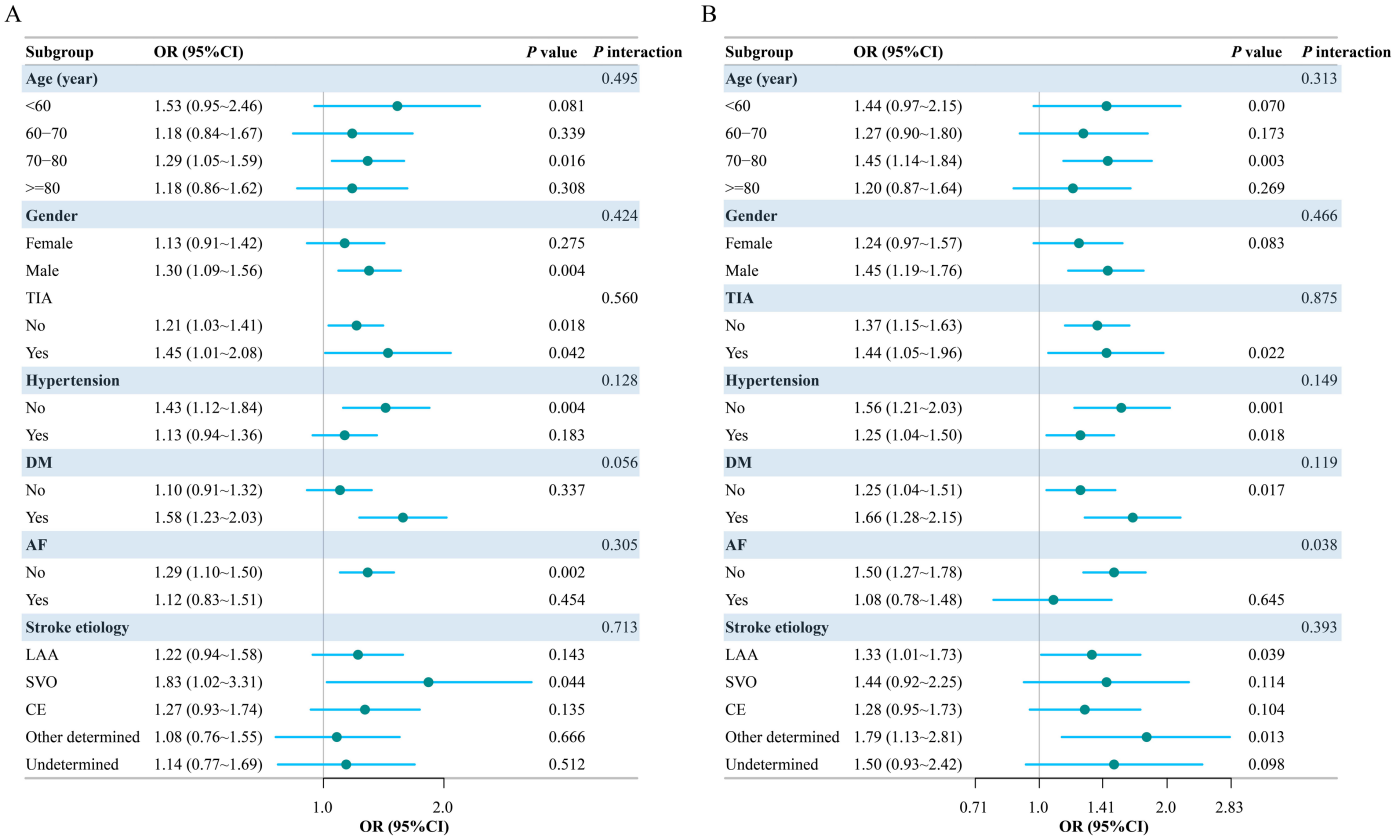
Subgroup and interaction analyses of the associations between CAR/CTI and 90-day unfavorable outcome. **(A)** Subgroup analysis for the association between CAR and the study outcome. **(B)** Subgroup analysis for the association between CTI and the study outcome. Odds ratios and 95% confidence intervals are shown for each subgroup. P values for interaction are presented on the right.

To assess robustness, prespecified subgroup analyses were performed (Figure 4). The association between CAR and 90-day unfavorable outcome was directionally consistent across strata defined by age, sex, vascular risk factors, and stroke etiology, with no evidence of effect modification (all *P* for interaction >0.05). Results for CTI were similarly consistent across most subgroups (all other interaction *p*-values >0.05); however, heterogeneity was observed by atrial fibrillation status (*P* for interaction = 0.038). CTI was associated with unfavorable outcome among patients without atrial fibrillation (OR 1.50, 95% CI 1.27–1.78), whereas no significant association was detected among those with atrial fibrillation (OR 1.08, 95% CI 0.78–1.48).

### 3.6 ROC analysis for predicting 90-day unfavorable outcomes after AIS

**Figure 5:**
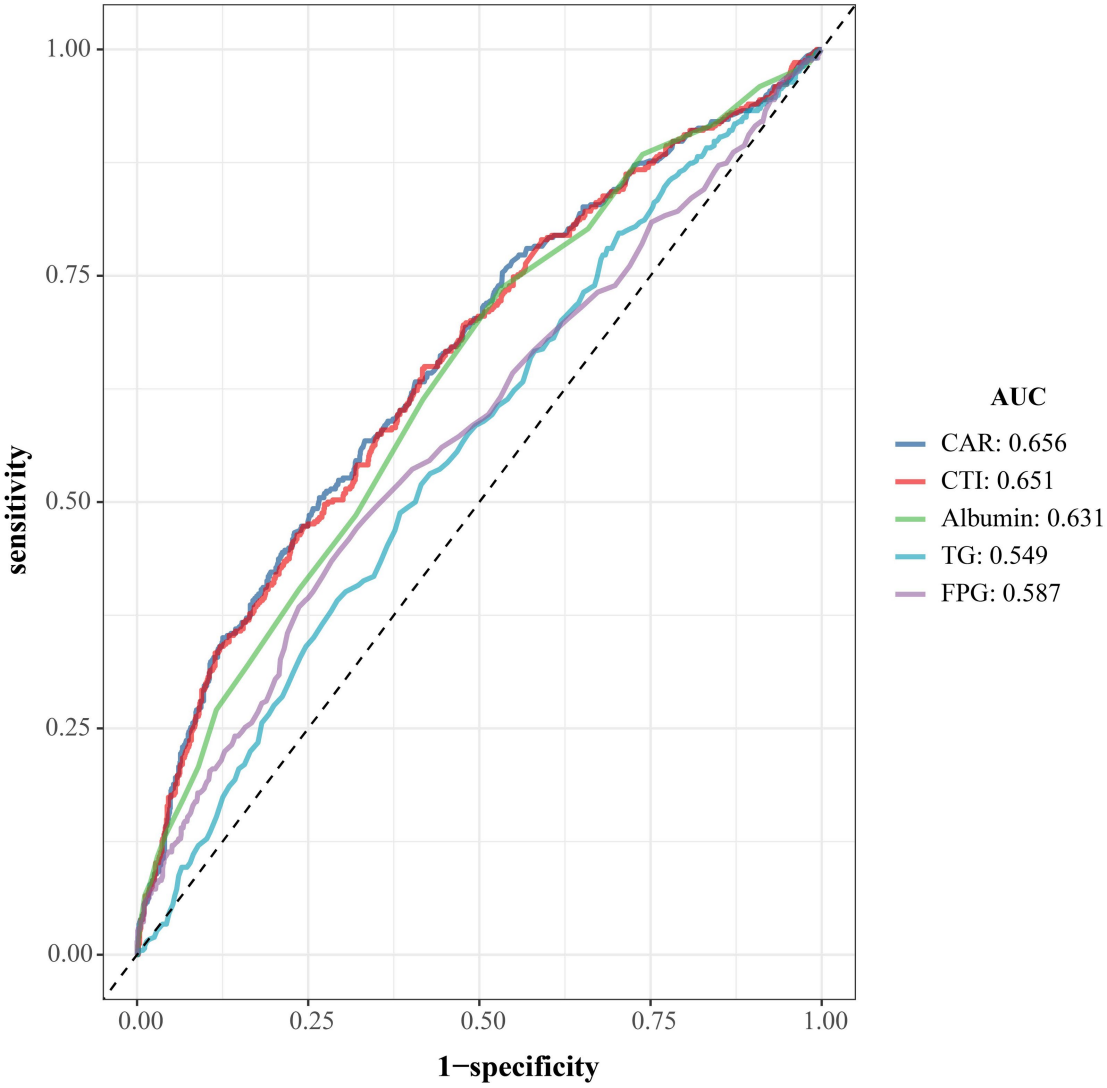
ROC curves comparing discrimination of CAR, CTI, and conventional laboratory markers for 90-day unfavorable outcome.

As shown in Figure 5, ROC curves were used to compare the discriminative performance of CAR, CTI, ALB, TG, and FPG for 90-day unfavorable outcome in AIS. CAR achieved the largest AUC (0.656), marginally exceeding CTI (0.651), and both outperformed ALB (0.631), FPG (0.587), and TG (0.549). Overall, CAR and CTI provided the strongest discrimination among the evaluated markers, with a slight advantage for CAR.

### 3.7 Incremental predictive value of CAR and CTI

To quantify the added prognostic contribution of CAR and CTI beyond the base model, we calculated the NRI and IDI (Table 4). Adding either marker improved both reclassification and discrimination. Specifically, inclusion of CAR yielded an NRI of 0.148 (95% CI 0.043–0.254) and an IDI of 0.010 (95% CI 0.004–0.017). The corresponding gains were larger after adding CTI (NRI 0.258, 95% CI 0.146–0.371; IDI 0.015, 95% CI 0.007–0.022; all *p* < 0.001). Overall, both indices provided incremental information, with CTI showing a greater improvement in point estimates.

**Table 4:** Incremental value of CAR and CTI in prediction.

Event	NRI (95% CI)	NRI *P*-value	IDI (95% CI)	IDI *P*-value
mRS				
Basic model	Ref	–	Ref	–
Basic model + CAR	0.148 (0.043–0.254)	0.006	0.010 (0.004–0.017)	0.003
Basic model + CTI	0.258 (0.146–0.371)	<0.001	0.015 (0.007–0.022)	<0.001

Basic Model: This model includes variables of PLT, TC, TG, HDL, BMI, HT, DM, CHD, Smoking, Initial NIHSS score. Abbreviations: NRI: Net Reclassification Improvement; IDI: Integrated Discrimination Improvement; CTI, C-reactive protein-triglyceride-glucose index; CAR, C-reactive protein/albumin ratio; BMI, body mass index; HT, hypertension; DM, diabetes mellitus; CHD, coronary heart disease; NIHSS, National Institutes of Health Stroke Scale score; PLT, platelets; HDL-C, high-density lipoprotein cholesterol; TC, total cholesterol; TG, triglycerides.

### 3.8 Penalized regression–based model development

#### 3.8.1 LASSO feature selection and model inputs

**Figure 6:**
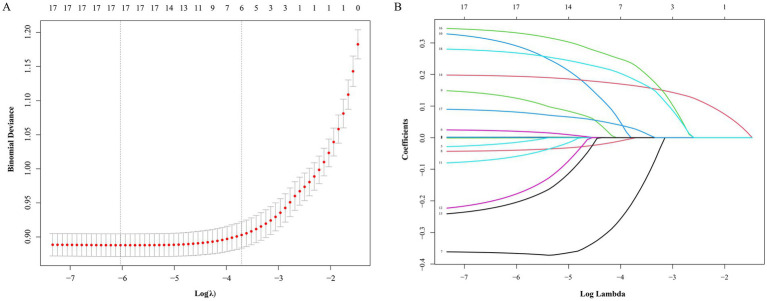
LASSO regression for predictor selection: cross-validated deviance and coefficient paths. **(A)** Cross-validation plot for selecting the optimal value of the tuning parameter lambda. **(B)** Coefficient profiles of candidate variables generated by LASSO regression.

Starting from the set of clinical covariates specified in Model 3, we used penalized regression with the LASSO to screen variables associated with 90-day unfavorable outcome after AIS (Figure 6). This approach applies an L1 penalty to compress regression weights, effectively excluding less informative predictors and reducing overlap among correlated features, which supports more stable out-of-sample performance. With the penalty parameter tuned to its optimal value (*λ*), the final subset comprised six variables: sex, age, BMI, admission NIHSS, CAR, and CTI.

#### 3.8.2 Model performance and clinical utility

**Figure 7:**
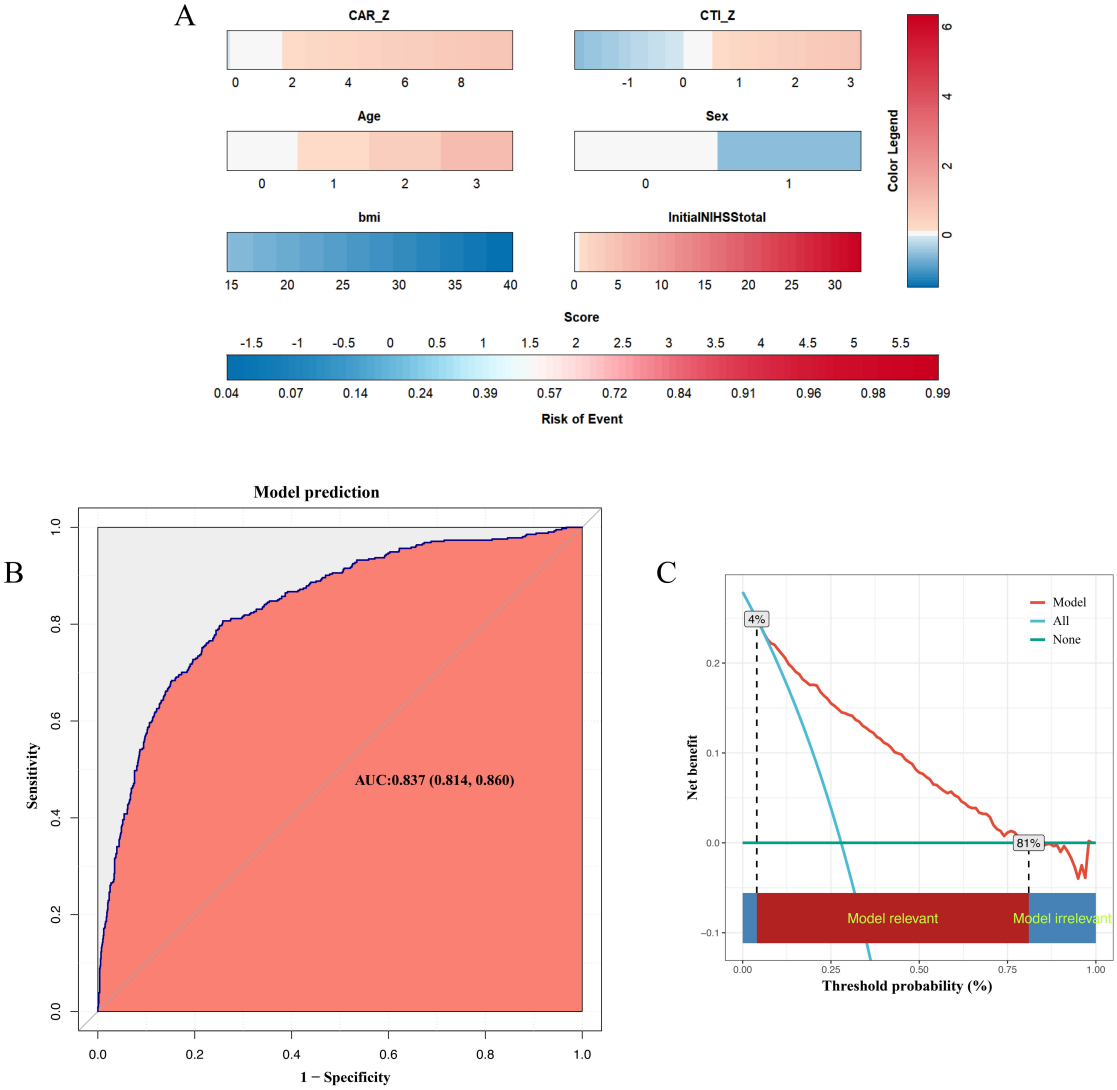
Predictive performance and clinical utility of the six-variable prognostic model: risk scoring, ROC curve, and decision-curve analysis. **(A)** Nomogram for individualized prediction of the study outcome. **(B)** Receiver operating characteristic curve showing the discriminative ability of the prediction model. **(C)** Decision curve analysis showing the clinical utility of the prediction model across different threshold probabilities.

Figure 7 summarizes the model presentation, discrimination, and clinical utility. Figure 7A visualizes the contribution of the six retained predictors (CAR, CTI, age, sex, BMI, and admission NIHSS) and provides an individualized estimate of the 90-day unfavorable outcome probability. The model showed good apparent discrimination (AUC 0.837, 95% CI 0.814–0.860; Figure 7B). Internal validation using repeated 10-fold cross-validation showed stable performance, with a mean cross-validated AUC of 0.831, mean Brier score of 0.142, calibration intercept of 0.023, and calibration slope of 1.018 (Supplementary Table S4). The apparent calibration plot also showed acceptable agreement between predicted and observed risks (Supplementary Figure S1), and the distribution of performance metrics across validation runs is shown in Supplementary Figure S2. In the decision-curve framework, the model yielded higher net benefit than treating all or treating none across a broad range of threshold probabilities (approximately 0.04–0.81; Figure 7C), although no prespecified clinical decision threshold was defined in the present study.

## 4 Discussion

To our knowledge, this study provides the first comprehensive evaluation of the independent prognostic utility of CAR and CTI for 90-day functional outcomes after AIS. The findings indicate that inflammation-related nutritional and metabolic composite indices are clinically informative for early risk stratification after AIS. Both CAR and CTI were independently associated with 90-day unfavorable functional outcome after extensive adjustment, supporting an inflammation–reserve–metabolism axis as a relevant prognostic dimension. A non-linear pattern was observed for CAR, whereas CTI showed a largely linear gradient and yielded greater incremental improvement in reclassification metrics when added to a conventional clinical model. Because the shape of the CAR association appeared sensitive to the distribution of observations across its range, this non-linear finding should be interpreted cautiously. A compact model derived using penalized regression, incorporating sex, age, BMI, admission NIHSS, CAR, and CTI, showed good apparent discrimination and favorable net benefit across a wide range of decision thresholds, suggesting potential value for early prognostic assessment. However, because the model was derived from a single-center secondary dataset and underwent internal validation only, it should currently be regarded as exploratory until external validation becomes available.

The CAR has been linked to adverse prognosis in multiple malignancies, including lung adenocarcinoma, oral cancer, and gastric cancer (28–31). In the stroke literature, higher CAR has been associated with mortality in AIS (15, 32), but its relationship with short-term functional disability has remained less clearly characterized. In the present analysis, CAR showed evidence of a non-linear association with 90-day unfavorable outcome. In the exploratory two-piecewise model, the association appeared more evident below the fixed breakpoint of 1.32, whereas the estimate above this point was less precise and did not reach statistical significance. Because relatively few patients were in the higher CAR range, this part of the curve should be interpreted cautiously. Overall, the findings support a non-linear relationship between CAR and outcome, but the exact shape of the association, particularly at higher CAR levels, requires confirmation in independent datasets. One possible explanation is that CAR reflects not only inflammatory burden but also aspects of physiological reserve related to albumin, which has anti-inflammatory, antioxidant, and endothelial-protective properties (33, 34). However, the biological meaning of the observed non-linear pattern remains uncertain, and the present findings should not be interpreted as establishing a clinically actionable threshold.

The CTI is designed to capture inflammatory activity together with metabolic dysregulation (35). Its strong linear association with unfavorable outcome in our analysis further supports the concept that inflammation–metabolic coupling is central to post-stroke pathophysiology (36, 37). Low-grade inflammation, insulin resistance, and lipid abnormalities often reinforce one another and may impede recovery through multiple pathways, including accelerated vascular injury, amplified ischemic damage, and reduced neuroplasticity (38–41). Because the CTI weighting scheme was adopted from prior work rather than derived within the present AIS cohort, some degree of population-specific miscalibration is possible. Although CTI showed prognostic value in the current analysis, future external validation studies should examine whether recalibration or re-estimation of the component weights improves performance in independent stroke populations. The finding in patients with atrial fibrillation should be interpreted cautiously. In our analysis, the association between CTI and unfavorable outcome appeared weaker in patients with AF than in those without AF, but subgroup analyses are exploratory and the estimates within individual strata are less stable. One possible explanation is that cardioembolic stroke may be driven more by thromboembolic and occlusion-related factors, whereas systemic inflammatory and metabolic disturbance may play a larger role in other stroke mechanisms (42–46). Even so, the present data are not sufficient to support a definite subgroup-specific interpretation, and this finding should be confirmed in independent datasets.

From a practical standpoint, both CAR and CTI can be derived from routine admission laboratory tests at relatively low cost, making them useful for early risk stratification, particularly in settings with limited resources. Because these indices rely on laboratory results and simple calculation, they are better viewed as tools for early prognostic assessment rather than immediate bedside evaluation. The incremental value observed for CTI indicates that incorporating it into established prediction frameworks based on variables such as NIHSS and age may improve discrimination and support more individualized prognostic counseling. In practice, once admission laboratory results and baseline clinical data are available, the model may help identify patients at higher risk of unfavorable outcome who may benefit from closer monitoring and further nutritional or metabolic assessment. However, no specific treatment threshold was defined in the present study, and the model should not be used as a substitute for clinical judgment. Although the six-variable model showed favorable internal performance, it should currently be viewed as exploratory rather than ready for routine clinical implementation.

This study has several strengths. It leveraged prospectively collected registry data with a relatively large sample and near-complete follow-up. In addition, a comprehensive analytic framework was implemented, combining multivariable adjustment, assessment of non-linear relationships, and data-driven modeling approaches, which together enhance the robustness and credibility of the findings. Formal multicollinearity diagnostics additionally did not suggest instability in the fully adjusted models, which supports the interpretability of the reported associations.

Several limitations should be acknowledged. First, the cohort was drawn from a single center, and both the patient case mix and local care pathways may have been institution-specific. Although internal validation suggested stable model performance, external validation in independent multicenter populations is still required before broader clinical use can be considered. Second, selection bias should be considered. A total of 600 of 2,084 initially screened patients (28.8%) were excluded, and the additional exclusions due to missing key laboratory data in the present secondary analysis may not have been random. Although remaining missing values among other covariates within the analytic cohort were handled using multiple imputation, exclusion of patients with missing variables required for CAR/CTI calculation may still have introduced bias. If such missingness was associated with stroke severity, care processes, or prognosis, the observed associations may have been affected. Third, despite extensive adjustment for measured covariates, residual confounding from unmeasured or incompletely captured factors cannot be fully ruled out. In particular, information on pre-stroke functional status, acute infection status, reperfusion therapies, and exact analyte-specific laboratory sampling time was not available or not sufficiently complete for consistent inclusion in the adjusted models. The E-values shown in Table 2 suggest that an unmeasured confounder would need to have at least moderate associations with both the exposure and the outcome to fully explain away the main findings. However, *E*-value analysis should be viewed as a sensitivity analysis rather than proof against residual confounding. Fourth, given the observational nature of the analysis, the findings are best interpreted as risk associations rather than causal effects. Fifth, the exact timing of blood collection relative to stroke onset was not available, and fasting status was not uniformly documented for the laboratory measures. Because several biochemical parameters may vary during the acute phase after ischemic stroke, residual variability related to sampling time and fasting condition may have influenced the observed associations. Finally, the study relied on baseline measurements and did not incorporate longitudinal CAR or CTI assessments, precluding evaluation of whether temporal trajectories of these indices provide additional prognostic information during follow-up.

Future studies should validate these findings in multicenter cohorts, evaluate whether CAR- or CTI-guided early nutritional or metabolic strategies can improve recovery, clarify performance across etiologic subtypes, and integrate imaging and broader biomarker layers to develop dynamic prediction tools with higher precision.

## 5 Conclusion

In this cohort of patients with AIS, admission CAR and CTI were independently associated with 90-day unfavorable functional outcome; CAR showed evidence of a non-linear association, whereas CTI was largely linear and provided greater incremental prognostic value. A compact model incorporating CAR and CTI improved prediction beyond conventional clinical factors and showed acceptable internal performance. However, the model should be considered exploratory, may serve only as an adjunct to early prognostic assessment, and requires external validation before clinical implementation.

## Data Availability

The original contributions presented in the study are included in the article/Supplementary material, further inquiries can be directed to the corresponding authors.
