# Supplementary material for: Associations of inflammation-related nutritional and metabolic status indices CAR and CTI with 90-day unfavorable functional outcomes in patients with acute ischemic stroke
Source: Front Nutr. 2026 Mar 23;13:1790922. doi: 10.3389/fnut.2026.1790922 (PMC13050733; doi:10.3389/fnut.2026.1790922)
Supplement: Supplementary file 1 [file Table_1.DOCX]

Supplementary Material

**Table S1** Multicollinearity diagnostics for covariates included in the fully adjusted CAR model.

**Table S2** Multicollinearity diagnostics for covariates included in the fully adjusted CTI model.

**Table S3** Descriptive comparison of baseline characteristics between the final analytic cohort and patients additionally excluded because of missing key laboratory variables.

**Table S4** Internal validation and calibration of the six-variable logistic prognostic model. Validation approach: repeated 10-fold cross-validation. Based on the exported results file, 50 validation runs were summarized. Apparent model metrics were taken from the model calibration output; cross-validation columns summarize the exported repeated-validation results.

**Fig. S1** Calibration plot of the six-variable logistic prognostic model.

**Fig. S2** Distribution of internal validation performance metrics across repeated 10-fold cross-validation runs for the six-variable logistic prognostic model

**Fig. S3** Distribution of CAR values in the analytic cohort, with the fixed breakpoint at 1.32 indicated.

| Supplementary Table S1. Multicollinearity diagnostics for covariates included in the fully adjusted CAR model. | | | | |
| --- | --- | --- | --- | --- |
|  |  |  |  |  |
| **Variables** | **GVIF** | **DF** | **GVIF^(1/(2*Df))** | **collinearity (0=No, 1= Yes)** |
| CAR | 1.160 | 1 | 1.077 | 0 |
| Sex | 1.628 | 1 | 1.276 | 0 |
| Age | 1.290 | 3 | 1.043 | 0 |
| PLT | 1.119 | 1 | 1.058 | 0 |
| BMI | 1.168 | 1 | 1.081 | 0 |
| DM | 1.375 | 1 | 1.173 | 0 |
| HT | 1.157 | 1 | 1.076 | 0 |
| Smoking | 1.570 | 1 | 1.253 | 0 |
| AF | 2.241 | 1 | 1.497 | 0 |
| InitialNIHSStotal | 1.213 | 1 | 1.101 | 0 |
| Scr | 1.103 | 1 | 1.050 | 0 |
| TG | 1.363 | 1 | 1.168 | 0 |
| TC | 1.488 | 1 | 1.220 | 0 |
| HDL-C | 1.440 | 1 | 1.200 | 0 |
| CHD | 1.068 | 1 | 1.033 | 0 |
| HbA1c | 1.295 | 1 | 1.138 | 0 |
| Ischemicstroke | 2.717 | 5 | 1.105 | 0 |
| Abbreviations: GVIF, Generalized variance inflation factor; DF, degree of freedom; CTI, C-reactive protein-triglyceride-glucose index; CAR, C-reactive protein/albumin ratio; BMI, body mass index; DM, diabetes mellitus; AF, atrial fibrillation; CHD, coronary heart disease; NIHSS, National Institutes of Health Stroke Scale score; PLT, platelets; HBA1c, hemoglobin Alc; HDL-C, high- density lipoprotein cholesterol; TC, total cholesterol; TG, triglycerides. | | | | |
|  |  |  |  |  |
|  |  |  |  |  |
|  |  |  |  |  |
|  |  |  |  |  |
|  |  |  |  |  |

| **Supplementary Table S2**. Multicollinearity diagnostics for covariates included in the fully adjusted CTI model. | | | | |
| --- | --- | --- | --- | --- |
|  |  |  |  |  |
| **Variables** | **GVIF** | **DF** | **GVIF^(1/(2*Df))** | **collinearity (0=No, 1= Yes)** |
| CTI | 1.169 | 1 | 1.081 | 0 |
| Sex | 1.631 | 1 | 1.277 | 0 |
| Age | 1.301 | 3 | 1.045 | 0 |
| PLT | 1.118 | 1 | 1.058 | 0 |
| BMI | 1.168 | 1 | 1.081 | 0 |
| DM | 1.378 | 1 | 1.174 | 0 |
| HT | 1.160 | 1 | 1.077 | 0 |
| Smoking | 1.575 | 1 | 1.255 | 0 |
| AF | 2.239 | 1 | 1.496 | 0 |
| InitialNIHSStotal | 1.229 | 1 | 1.109 | 0 |
| Scr | 1.105 | 1 | 1.051 | 0 |
| TG | 1.371 | 1 | 1.171 | 0 |
| TC | 1.487 | 1 | 1.219 | 0 |
| HDL-C | 1.440 | 1 | 1.200 | 0 |
| CHD | 1.067 | 1 | 1.033 | 0 |
| HbA1c | 1.292 | 1 | 1.137 | 0 |
| Ischemicstroke | 2.718 | 5 | 1.105 | 0 |
| Abbreviations: GVIF, Generalized variance inflation factor; DF, degree of freedom; CTI, C-reactive protein-triglyceride-glucose index; CAR, C-reactive protein/albumin ratio; BMI, body mass index; DM, diabetes mellitus; AF, atrial fibrillation; CHD, coronary heart disease; NIHSS, National Institutes of Health Stroke Scale score; PLT, platelets; HBA1c, hemoglobin Alc; HDL-C, high- density lipoprotein cholesterol; TC, total cholesterol; TG, triglycerides. | | | | |
|  |  |  |  |  |
|  |  |  |  |  |
|  |  |  |  |  |
|  |  |  |  |  |
|  |  |  |  |  |

| **Supplementary Table S3.** Descriptive comparison of baseline characteristics between the final analytic cohort and patients additionally excluded because of missing key laboratory variables. | | |
| --- | --- | --- |
|  |  |  |
| **Variables** | **Included in final analysis (n = 1484)** | **Additionally excluded due to missing key laboratory variables (n = 422)** |
| **Demographics** |  |  |
| Sex (Male, n (%)) | 912 (61.5) | 256 (60.66) |
| Age, n (%) |  |  |
| <60 | 324 (21.8) | 112 (26.54) |
| 60-70 | 393 (26.5) | 112 (26.54) |
| 70-80 | 528 (35.6) | 142 (33.65) |
| >=80 | 239 (16.1) | 56 (13.27) |
| BMI, Mean ± SD | 23.48 ± 3.28 | 23.56 ± 3.16 |
| Smoking, n (%) | 599 (40.4) | 111 (26.30) |
| **Medical history, n (%)** |  |  |
| Hyperlipidemia | 551 (37.1) | 148 (35.07) |
| DM | 456 (30.7) | 158 (37.44) |
| AF | 312 (21.0) | 95 (22.51) |
| CHD | 175 (11.8) | 45 (10.66) |
| TIA | 308 (20.8) | 94 (22.27) |
| **Clinical status on admission** |  |  |
| Initial NIHSS score Median (IQR) | 3.0 (1.0, 7.0) | 4.00 (2.00, 7.00) |
| Discharge NIHSS, Median (IQR) | 2.0 (0.0, 4.0) | 2.00 (0.00, 4.00) |
| Stroke etiology, n (%) |  |  |
| LAA | 490 (33.0) | 120 (28.44) |
| SVO | 283 (19.1) | 82 (19.43) |
| CE | 373 (25.1) | 116 (27.49) |
| Other determined | 124 ( 8.4) | 47 (11.14) |
| Undetermined | 213 (14.4) | 57 (13.51) |
| NRI, Mean ± SD | 105.21 ± 9.66 | 104.87 ± 9.87 |
| **Nutritional risk index, n (%)** |  |  |
| No risk | 1083 (73.0) | 15 ( 3.55) |
| Mild risk | 92 ( 6.2) | 62 (14.69) |
| Moderate risk | 278 (18.7) | 30 ( 7.11) |
| Severe risk | 31 ( 2.1) | 315 (74.64) |
| **Laboratory parameters** |  |  |
| WBC (10^9/L, Mean ± SD) | 8.20 ± 2.96 | 7.90 ± 2.59 |
| RBC (10^9/L, Mean ± SD) | 4.33 ± 0.63 | 4.28 ± 0.67 |
| HGB(g/L, Mean ± SD) | 13.50 ± 1.98 | 13.40 ± 2.08 |
| RDW (%, Mean ± SD) | 13.38 ± 1.53 | 13.44 ± 1.56 |
| PLT (10^9/L, Mean ± SD) | 225.24 ± 69.79 | 217.91 ± 76.24 |
| TC (mg/dL, Mean ± SD) | 180.66 ± 43.95 | 174.75 ± 42.85 |
| TG (mg/dL, Mean ± SD) | 109.71 ± 54.58 | 117.53 ± 62.79 |
| HDL-C (mg/dL, Mean ± SD) | 46.63 ± 13.59 | 35.48 ± 23.04 |
| LDL-C (mg/dL, Mean ± SD) | 108.83 ± 38.39 | 87.73 ± 50.95 |
| BUN(ummol/L, Mean ± SD) | 17.46 ± 8.89 | 18.10 ± 8.85 |
| Creatinine (mg/dL, Median (IQR)) | 0.9 (0.7, 1.1) | 0.91 (0.75, 1.10) |
| ALB (g/dL, Mean ± SD) | 4.03 ± 0.42 | 3.99 ± 0.46 |
| Total protein (g/dL, Mean ± SD) | 7.02 ± 0.59 | 6.97 ± 0.68 |
| FPG (mg/dL, Mean ± SD) | 106.58 ± 38.57 | 88.80 ± 53.17 |
| HbA1c(%, Mean ± SD) | 5.24 ± 2.64 | 5.75 (0.00, 6.40) |
| CRP (mg/L, Median (IQR)) | 1.6 (0.6, 5.3) | 0.20 (0.05, 1.10) |
| Abbreviation: CTI, C-reactive protein-triglyceride-glucose index; BMI, body mass index; DM, diabetes mellitus; AF, atrial fibrillation; CHD, coronary heart disease; NIHSS, National Institutes of Health Stroke Scale score; TIA transient ischemic attack; LAA, large artery atherosclerosis; SVO, small vessel occlusion; CE, cardiac embolism; NRI, nutritional risk index; WBC, white blood cell; RBC, red blood cell; HGB, hemoglobin; RDW, red cell distribution width; PLT, platelets; ALB, albumin; FPG, fasting plasma glucose; HBA1c, hemoglobin Alc; HDL-C, high- density lipoprotein cholesterol; LDL-C, low-density lipoprotein cholesterol; CRP, C-reactive protein; TC, total cholesterol; BUN, blood urea nitrogen; TG, triglycerides. P - value less than 0.05 is expressed in bold. | | |
|  |  |  |
|  |  |  |
|  |  |  |
|  |  |  |
|  |  |  |
|  |  |  |
|  |  |  |

| **Supplementary Table S4.** Internal validation and calibration of the six-variable logistic prognostic model. Validation approach: repeated 10-fold cross-validation. Based on the exported results file, 50 validation runs were summarized. Apparent model metrics were taken from the model calibration output; cross-validation columns summarize the exported repeated-validation results. | | | | | | |
| --- | --- | --- | --- | --- | --- | --- |
|  |  |  |  |  |  |  |
|  |  |  |  |  |  |  |
| **Metric** | **Apparent model** | **Cross-validation mean** | **SD across runs** | **Median** | **Min** | **Max** |
| **C (ROC)** | 0.837 | 0.831 | 0.037 | 0.841 | 0.754 | 0.924 |
| **Brier** | 0.140 | 0.142 | 0.017 | 0.138 | 0.108 | 0.178 |
| **Intercept** | 0.000 | 0.023 | 0.389 | -0.003 | -0.699 | 1.188 |
| **Slope** | 1.000 | 1.018 | 0.236 | 1.031 | 0.610 | 1.718 |
| **Emax** | 0.108 | 0.181 | 0.093 | 0.159 | 0.057 | 0.502 |
| **E90** | 0.069 | 0.117 | 0.051 | 0.108 | 0.032 | 0.241 |
| **Eavg** | 0.029 | 0.050 | 0.016 | 0.050 | 0.012 | 0.080 |
| **R2** | 0.379 | 0.356 | 0.098 | 0.382 | 0.111 | 0.545 |
| Abbreviations: ROC, receiver operating characteristic; Emax, maximum calibration error; E90, 90th percentile calibration error; Eavg, average calibration error. Ideal values: higher C (ROC) and R2 indicate better discrimination/explained variation; lower Brier, Emax, E90, and Eavg indicate better overall performance/calibration; calibration intercept near 0 and slope near 1 indicate good calibration. | | | | | | |
|  |  |  |  |  |  |  |
|  |  |  |  |  |  |  |
|  |  |  |  |  |  |  |
|  |  |  |  |  |  |  |


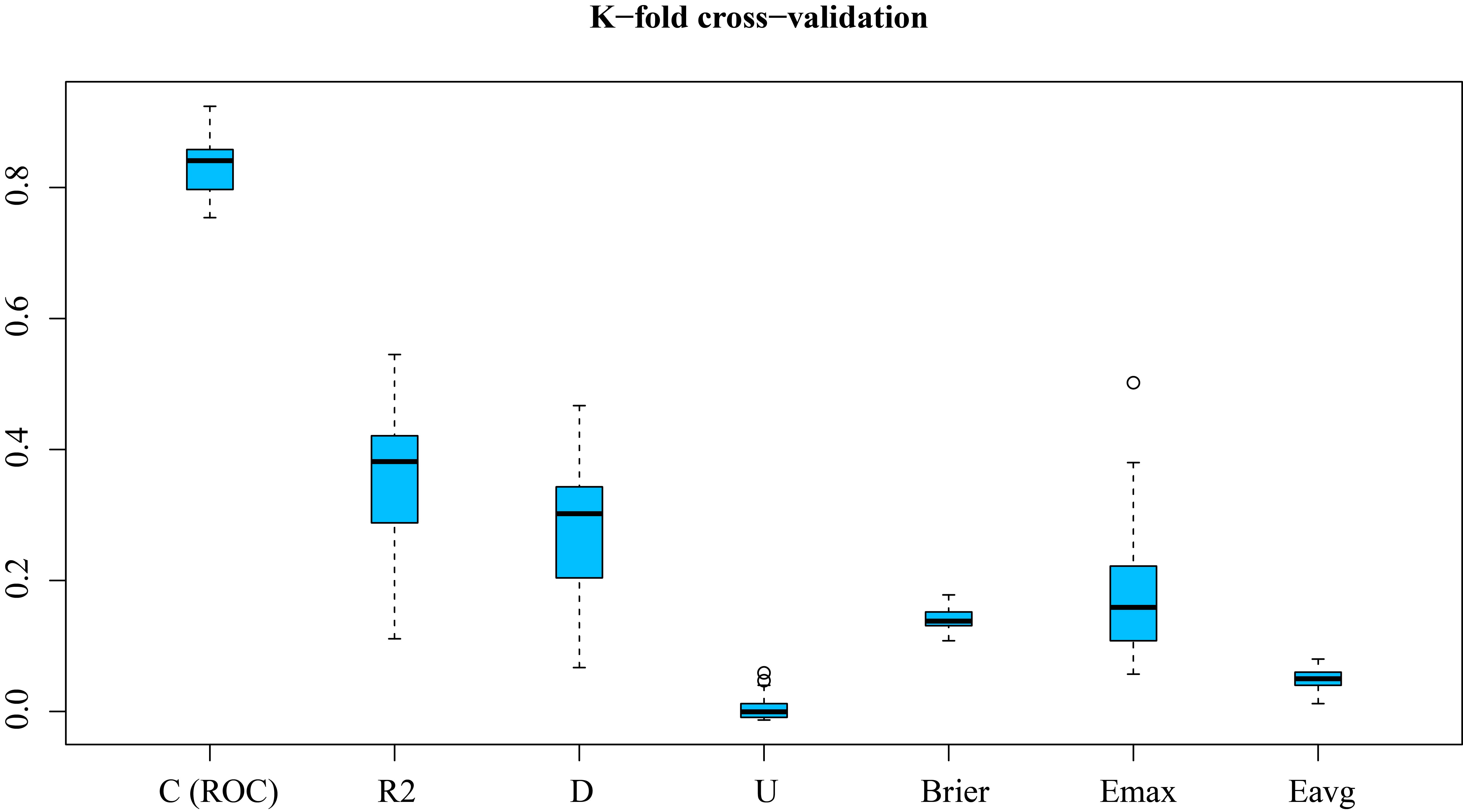


**Supplementary Figure S1.** Calibration plot of the six-variable logistic prognostic model.


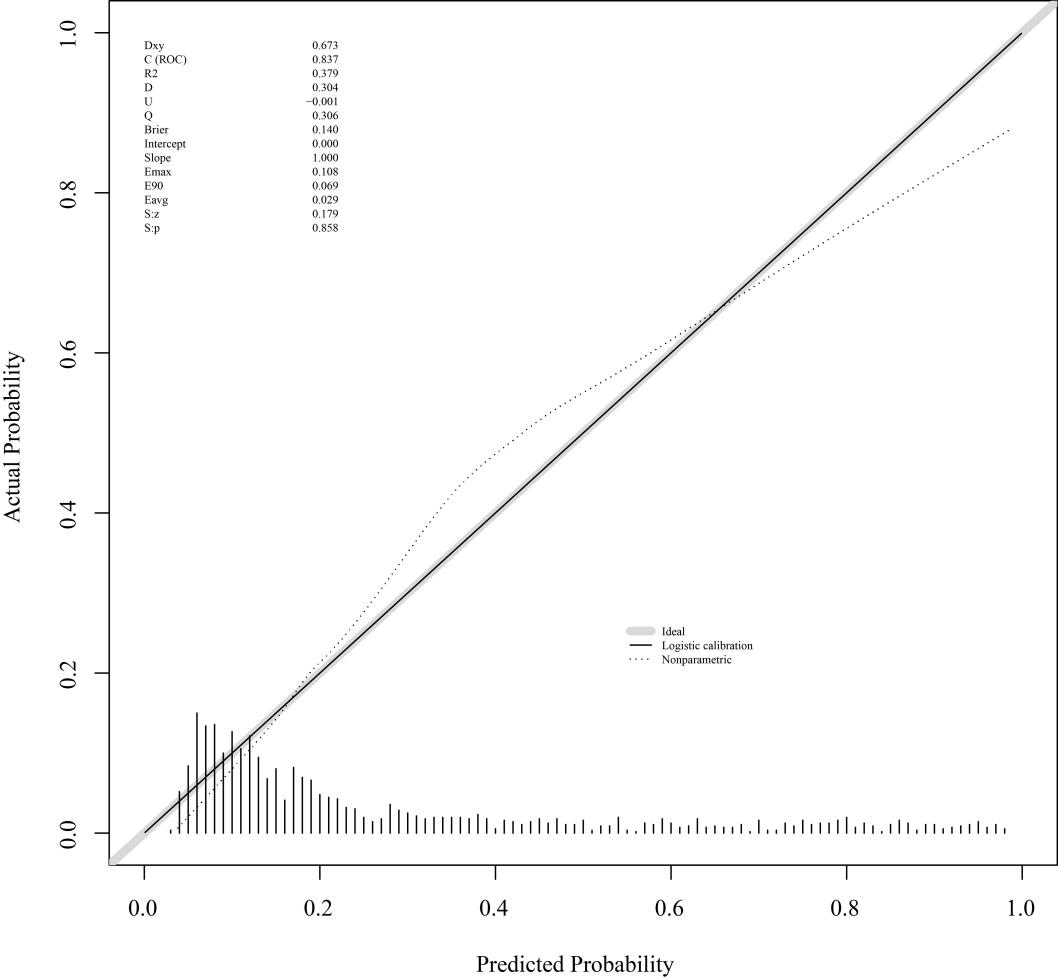


**Supplementary Figure S2.** Distribution of internal validation performance metrics across repeated 10-fold cross-validation runs for the six-variable logistic prognostic model


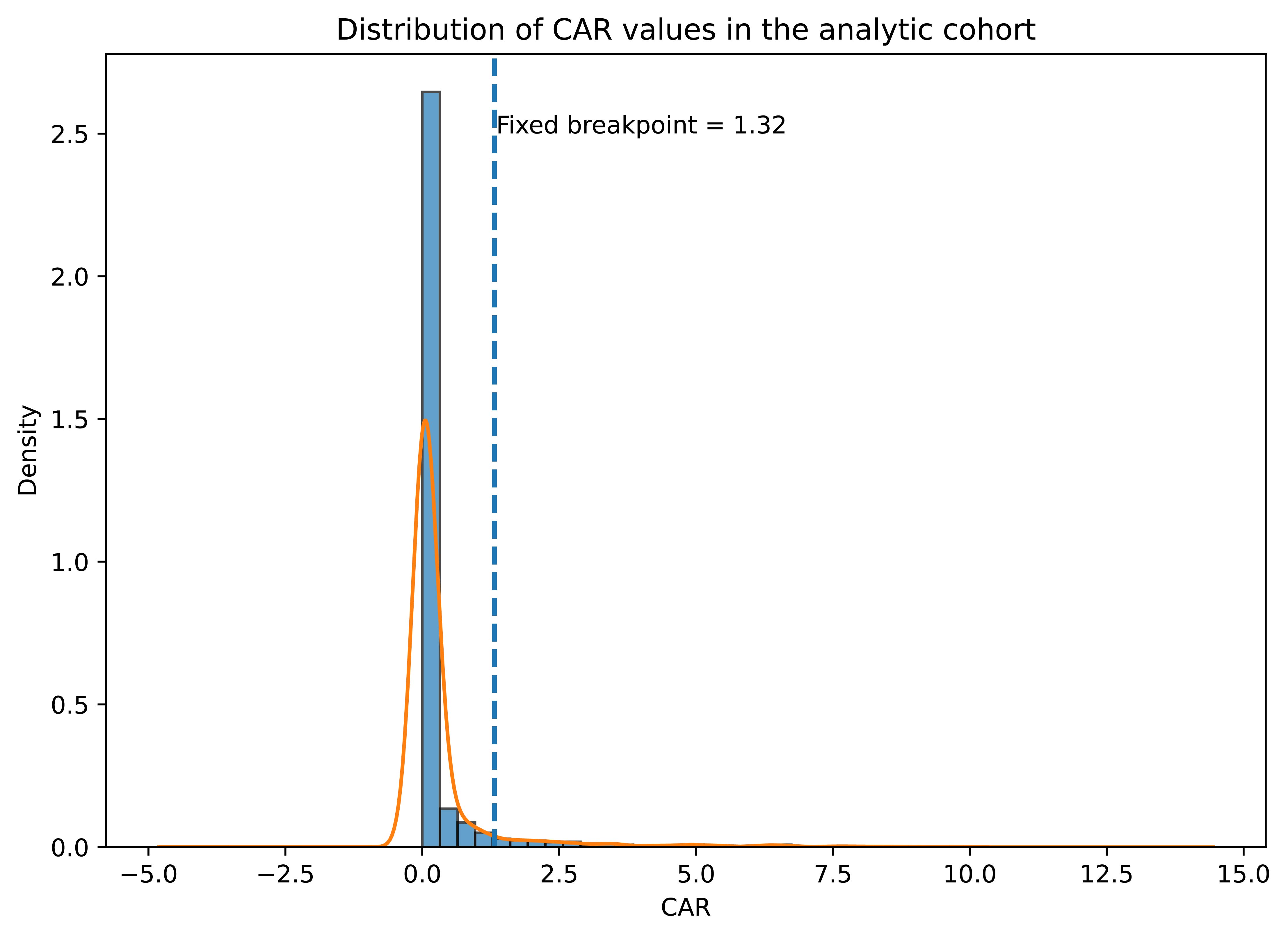


**Supplementary Figure S3.** Distribution of CAR values in the analytic cohort, with the fixed breakpoint at 1.32 indicated.
